# Facial expression recognition using three-stage support vector machines

**DOI:** 10.1186/s42492-019-0034-5

**Published:** 2019-12-16

**Authors:** Issam Dagher, Elio Dahdah, Morshed Al Shakik

**Affiliations:** Computer Engineering Department, University of Balamand, Tripoli, P.O.BOX 100 Lebanon

**Keywords:** Facial expression recognition, Support vector machine, Histogram of oriented gradients, Viola–Jones, Validation

## Abstract

Herein, a three-stage support vector machine (SVM) for facial expression recognition is proposed. The first stage comprises 21 SVMs, which are all the binary combinations of seven expressions. If one expression is dominant, then the first stage will suffice; if two are dominant, then the second stage is used; and, if three are dominant, the third stage is used. These multilevel stages help reduce the possibility of experiencing an error as much as possible. Different image preprocessing stages are used to ensure that the features attained from the face detected have a meaningful and proper contribution to the classification stage. Facial expressions are created as a result of muscle movements on the face. These subtle movements are detected by the histogram-oriented gradient feature, because it is sensitive to the shapes of objects. The features attained are then used to train the three-stage SVM. Two different validation methods were used: the leave-one-out and K-fold tests. Experimental results on three databases (Japanese Female Facial Expression, Extended Cohn-Kanade Dataset, and Radboud Faces Database) show that the proposed system is competitive and has better performance compared with other works.

## Introduction

Artificial intelligence has created higher standards for innovation and has introduced new possibilities for human–computer interaction. The goal of achieving communication between computers and humans is now a possibility; however, 55%–94% of human communication is nonverbal [1]. Thus, there is a need to develop an accurate facial expression recognition (FER) system that can establish efficient communication between humans and computers. The development of such a system can also be useful in several areas, such as lie detectors, surveillance, smart computing, visual development, computer gaming, and augmented reality [2].

For efficient communication to occur between two people, seven expressions are globally identified and analyzed to make communication smooth and reliable. Translating this idea into a computer system, the FER system can improve the way computers interact with humans and lead to further advancements in this field.

Many researchers have tried developing the optimal FER system. Generally, the main approaches were to do it via machine learning or deep learning. The majority of studies used the machine-learning approach, because deep learning is a recent trend. Also, deep learning requires a massive amount of computation time, speed, and memory, whereas machine learning does not have all these requirements. The possible combinations are endless, because any or all of the following can be changed: face detection, feature extraction, and classification. Some studies in which the method of feature extraction was changed, whereas the detection and classification were kept the same, are as follows. Shan et al. [3] introduced an FER system that can detect facial expressions using a boosted version of local binary patterns (LBPs) and classified it using support vector machines (SVMs). Shih et al. [4] used linear discriminant analysis (LDA) and SVM. Khan performed a LBP-pyramid with SVM classification [1]. Jaffar [1] performed LBP with a Gabor filter. All these methods are based on machine learning, where the face was detected as a whole, and then each model was implemented. Some researchers decided to segment the face rather than take it as a whole and changed their extraction method. Chen et al. [5] applied facial segmentation to separate the eyes and mouth from the face before applying the histogram-oriented gradients (HOG) feature and classifying the image using an SVM. Also, Chen et al. [5] used a patch-based Gabor filter after segmenting each component of the face. Others decided to combine several extraction methods and then speed up the computation time. Liu et al. [6] combined LBP and HOG methods for feature extraction; then, they performed principal component analysis (PCA) to minimize the time and speed needed for computation, and, finally, they used SVM classification. However, some decided to implement deep learning rather than continuing to use machine learning. Mollahosseini et al. [7] used a deep-neural-network system. The number of combinations is endless, as can be seen above; however, their accuracies are fairly competitive. Thus, many researchers have decided to modify images by using various preprocessing techniques — spatial orientation, histogram equalization, color conversion, resolution enhancement, and many others.

The technique applied in this study generally revolves around the preprocessing of the image before extracting its features and the stages of classification. The classification is split into three individual stages composed of binary comparisons where the system enters each stage depending on the necessity and the case.

### Related work

In a previous study [8], the authors of that work used histograms of gradients as a feature extraction method. Three-dimensional FER was analyzed in other research [9]. A dictionary-based approach for FER was used [10]. Gabor wavelets and learning vector quantization were used [11]. FER using contourlet transform was done [12]. Exploring shape deformation is done [13]. In other work [14], SVM was used in FER. A convolutional neural network (CNN) was used [15]. In another study [16], the authors of that report used pairwise feature selection in classification. Multiple CNNs were used in other research [17]. Prototype-based modeling for facial expression analysis was implemented [18]. Curvelet transform was used in FER [19]. A guide to recognizing emotions from facial clues was presented [20]. Analysis of FER with occlusions was done in other work [21]. Another study [22] emphasized the line-based caricatures in FER. FER using extended LBP (ELBP) based on covariance matrix transform in Karhunen–Loeve transform was utilized in ref. [23]. Other researchers [24] used 3-D facial feature distances. Automatic FER using features of salient facial patches was demonstrated [25]. Fisher discriminant analysis was evaluated [26]. Other researchers [27] employed segmentation of face regions in FER. In other work [28], a deep fusion CNN was used in FER. Also, 2dPCA was used [29]. Other researchers [30] used the LBP as a feature extraction method in FER.

## Contributions

- Three-level-network: This network consists of three stages, where the primary and first stage is made up of 21 SVMs, which are all the binary combinations of the seven expressions. If one expression is dominant, then the first stage suffices. If two are dominant, the second stage is needed. If three are dominant, the third stage is used.
- An image preprocessing stage is used to ensure that the features attained from the face detected have a meaningful and proper contribution to the classification stage.
- Experimental results on three databases, Japanese Female Facial Expression (JAFFE), Extended Cohn-Kanade Dataset (CK+), and Radboud Faces Database (RaFD), show that the proposed system is competitive and has better performance compared with other systems.

## General model and mathematical computation

**Fig. 1:**
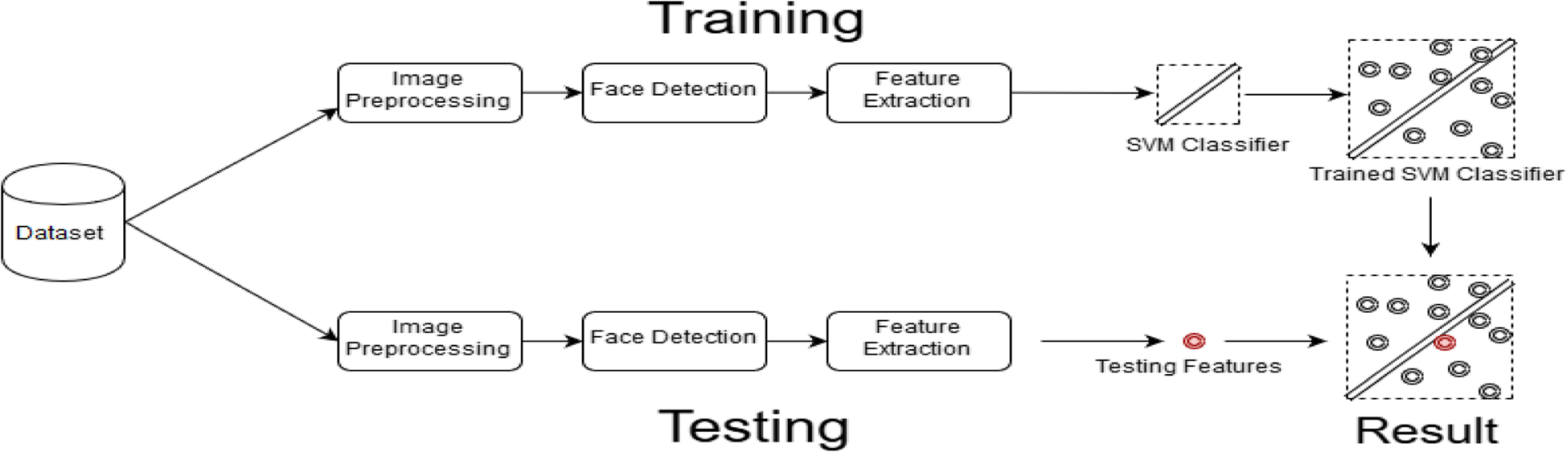
General skeletal structure: dividing the dataset into training and testing sets

The skeletal structure of the model is shown in Fig. 1. The images of the datasets are divided into the training set and testing set. The number of images in each set varies depending on what type of validation method is implemented. The images of each set undergo some preprocessing techniques, followed by facial detection and feature extraction. The features are then classified using SVM classification. Once the system is trained, the set to be tested undergoes a similar process to the trained set; however, ultimately, the features extracted from the image are compared with the SVM classifiers, and the result is then obtained.

**Fig. 2:**
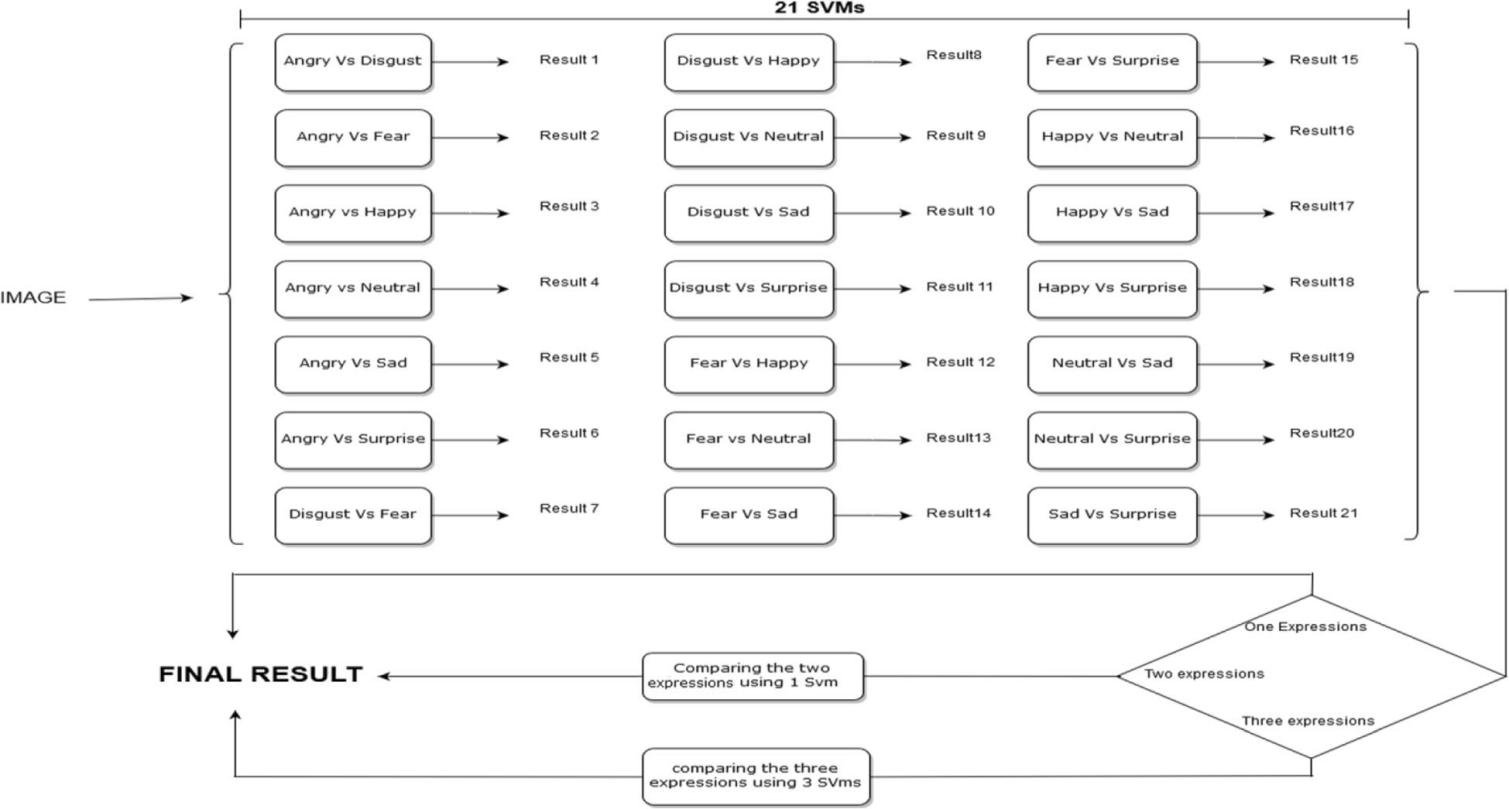
Two-stage model: first stage (21 SVM) and Second and third stages combining the results of Stage 1

Two-part model SVM classifiers are designed in such a way that they are made up of three stages (Fig. 2). Each stage represents a unique function to reach the final result. The first stage consists of a network of 21 SVMs. Because there are seven main expressions, each expression is compared with another expression, thus making a unique binary SVM. The total number of possible combinations resulting from this binary comparison is shown in Eq. (1), where selecting two expressions from the total number of expressions makes it possible to determine all possible binary outcomes.

1 $$ {C}_2^7=\frac{7!}{2!5!}=21 $$

(Combination 2 out of 7 expressions)

There are 21 different results from the SVMs shown above, and each expression has its own counter that counts the occurrences of the expression at the output of the 21 SVMs. After the occurrences have been calculated, the counters are compared with each other, and the result follows one of the following conditions.

- If one expression occurs the most at the outputs, then the final result definitely represents this expression.
- If two expressions equally occur at the outputs, the two expressions go into another stage, called “Stage 2”. Stage 2 has one SVM that is trained with these two expressions only and compared one last time to get a final result. The result of the SVM in Stage 2 represents the final result.
- If three expressions occur equally at the outputs, the three expressions enter another stage, called “Stage 3”. Similar to Stage 2, Stage 3 has three SVMs that represent all the possible combinations among the three expressions. The same concept of the 21 SVMs is applied, and the occurrences are counted. Then, the final result is determined from the output of this stage.

## Datasets and image preprocessing

**Fig. 3:**
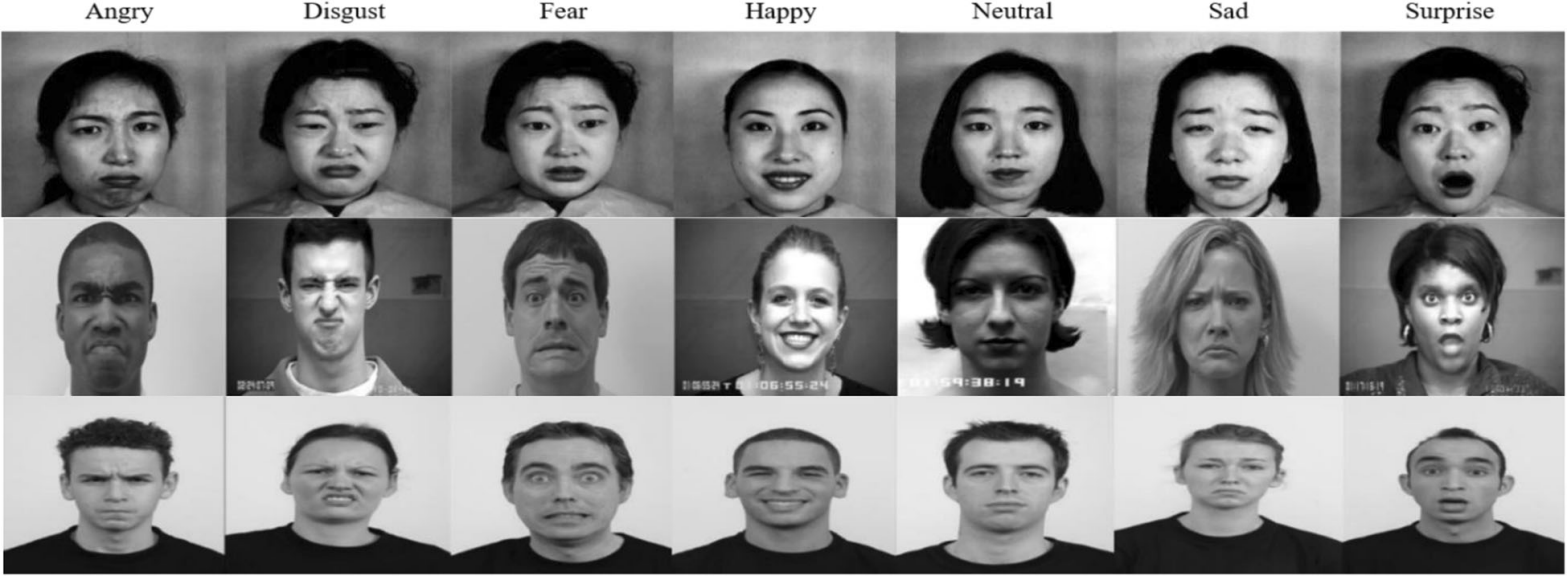
Sample of seven expressions of each dataset starting from JAFFE (top), CK+ (middle), and RaFD (bottom)

To evaluate the performance of the proposed approach, the system was tested on three commonly adopted datasets (Fig. 3): the JAFFE database [31], CK+ [32], and RaFD [33].

### JAFFE database

The dataset was taken from the Psychology Department at Kyushu University. This database consists of the seven primary expressions that were posed by 10 Japanese female models. The database consists of a total of 213 images, where 30 images are angry, 29 images are disgust, 32 images are fear, 31 images are happy, 30 images are neutral, 31 images are sad, and 30 images are surprise. Each model provided approximately three images for each facial expression. Each image was saved in grayscale with a resolution of 256 × 256 [31].

### CK+ database

This dataset consists of eight expressions (seven primary expressions plus contempt) that were posed by more than 200 adults ranging from 18 to 50 years of age. It generally consisted of Euro-American and Afro-American individuals. The images were taken in time frames where the initial frame was neutral, which was then transitioned into the expression that was desired at the end frame (the peak frame). These images were saved, some in grayscale and some in color, in 640 × 490 or 640 × 480 pixels. The database consists of 123 neutral images and 327 peak images (images with a certain expression). These 327 images consist of 45 images that are angry, 18 images that are contempt, 59 images that are disgust, 25 images that are fear, 69 images that are happy, 28 images that are sad, and 83 images that are surprise. The contempt expression images are excluded in this work [32].

### RaFD database

This dataset consists of 67 models: 20 male adults, 19 female adults, 4 male children, 6 female children, and 18 Moroccan male adults. Each model is pictured at a different gaze direction (left, frontal, and right) and at five different camera angles simultaneously. The dataset consists of eight expressions (seven primary expressions + contempt) and 1608 images that are distributed into 201 angry images, 201 contempt images, 201 disgust images, 201 fear images, 201 happy images, 201 neutral images, 201 sad images, and 201 surprise images with a resolution of 640 × 1024 pixels each. The frontal facing images (images at an angle of 90°) were worked on, and the contempt expression was excluded from this work [33].

### Image preprocessing

The datasets have different properties, such as resolution, size, and color. To unify the system to work on all datasets, standard image properties had to be developed.

**Fig. 4:**
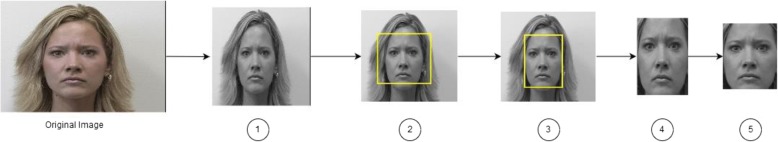
Image preprocessing: (1) Gray scaling and resizing, (2) Viola–Jones, (3) border adjustment, (4) cropping, and (5) additional resizing• Viola-Jones

As shown in Fig. 4, to reach the standard image input, the following steps were implemented:

- Gray scaling and resizing
- Viola–Jones detection
- Border adjustment
- Cropping
- Additional resizing

#### Gray scaling and resizing

**Fig. 5:**
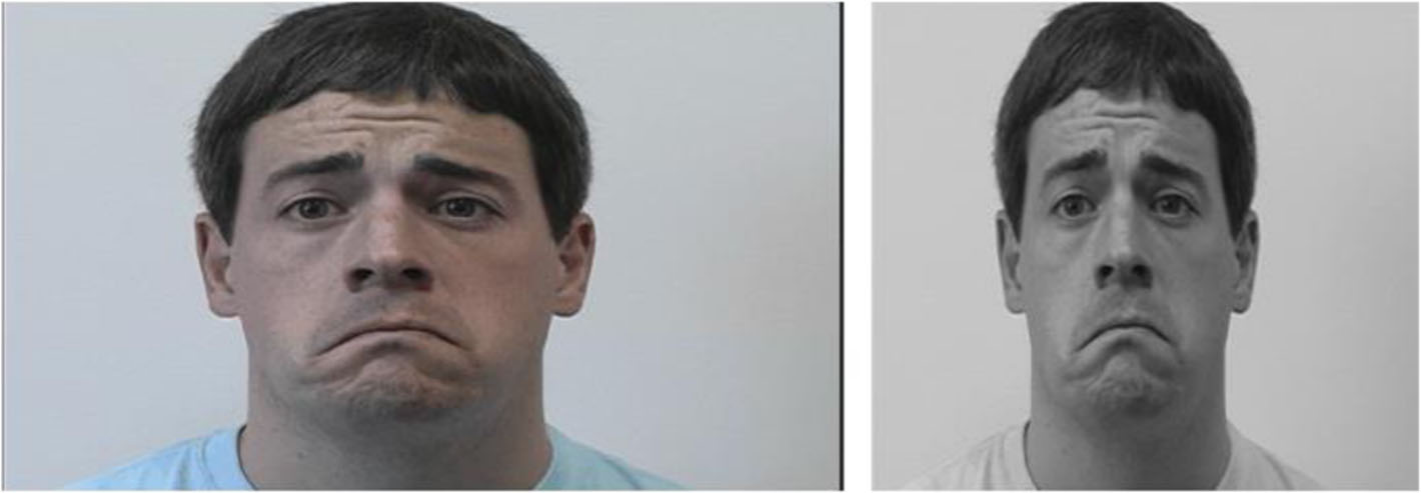
Grayscale and resizing to 256 × 256

The images in the JAFFE dataset were originally in grayscale format, whereas the images in the CK+ and the RaFD datasets had a mixture of color and grayscale images. Therefore, to unify the input type, the images were all tested, whether they were in grayscale format or not. If the images were colored, they were converted to gray. The next step was to ensure that all images were of equal size. The smallest dataset size was the JAFFE dataset, which included images of size 256 × 256. Therefore, images of CK+ and RaFD had to be resized to 256 × 256 to provide a standard input size. Fig. 5 shows the image after performing the grayscale conversion and the resizing. The resolution was not affected, and this not only unified the input size and color but also removed any color lamination that the image may have had.

#### Viola–Jones detection and border adjustment

**Fig. 6:**
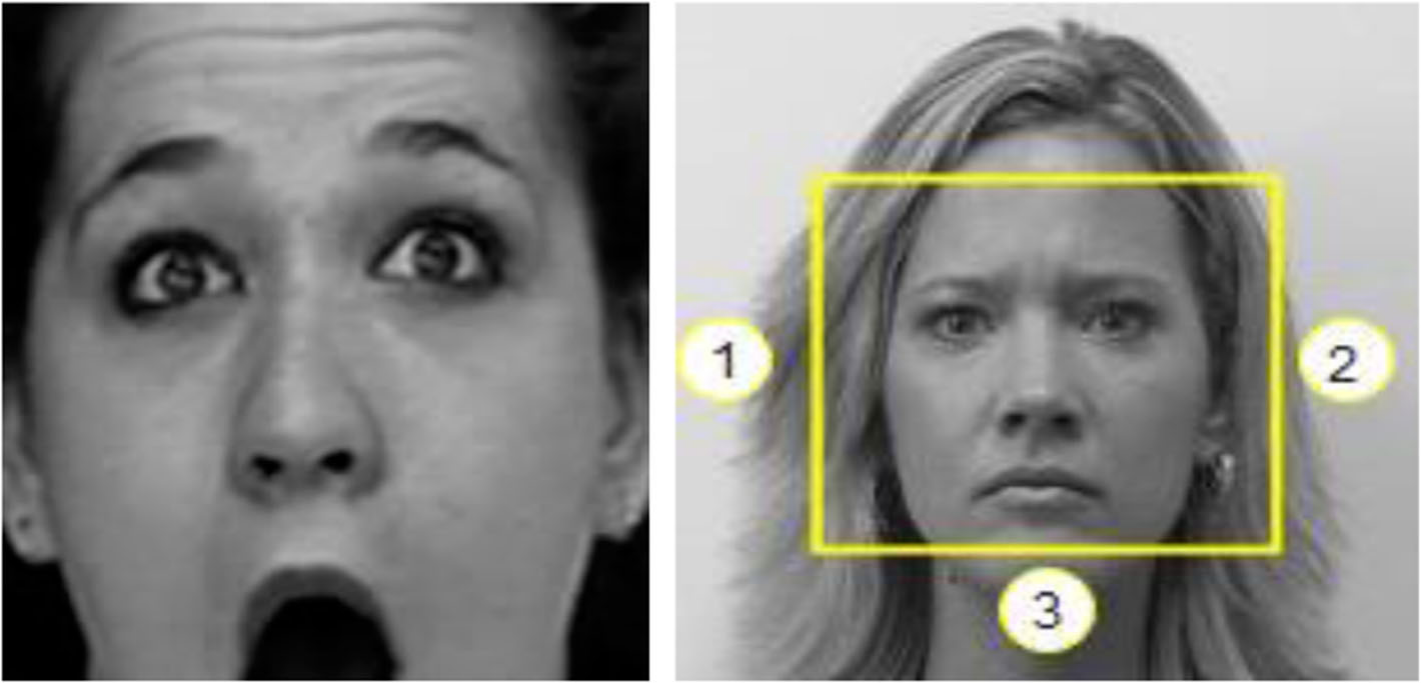
Viola–Jones face detection with mouth missing (left): Border 3 must cover the whole mouth only, whereas Borders 1 and 2 must cover the face region (right)

The Viola–Jones algorithm was implemented to detect the face from the image. Some modifications were performed to detect only one face from the image that is the clearest face. This was done by comparing the sizes of the “supposed” faces detected and then selecting the face with the largest dimensions. The Viola–Jones algorithm could capture the majority of the faces of the datasets accurately; however, it experienced some problems detecting faces displaying the surprise expression. The nature of the surprise expression is for the mouth to be wide open with raised eyebrows. As can be seen in Fig. 6 (left), the mouth is cropped, which leads to false classification in the system. Also, extra components were detected at the edges of the face, which also leads to false classifications.

#### Border adjustment

Border adjustment was a reasonable solution to these problems. Fig. 6 (right) displays the possible three borders that must be varied to give proper results. To optimize the input image, Border 3 must cover the whole mouth only, whereas Borders 1 and 2 must cover the face region only, without the ears. The optimal value for the extension of Border 3 is 8 pixels downward. Values smaller than this did not solve the problem of the cropped mouth, whereas values larger than this began to cover the chin and neck of the person, which introduced new unnecessary features that increase the chance of false classification. Regarding Borders 1 and 2, the borders had to be moved inward to remove any unnecessary details that can cause confusion to the system. The optimal values for Borders 1 and 3 were 20 and 20 pixels, respectively. A value lower than this is futile, because the ears are still covered within the borders, whereas a value higher than this is very bad, because the borders crop parts of the eyes.

#### Cropping

One border was left unmodified: the top border. The options here were either to crop the image, including the forehead of the face, or to crop it without the forehead. This choice had to be determined by testing, because the forehead was a point of confusion, and it was not clear whether the forehead contributes to the expression or is just unnecessary.

The system was tested once with the forehead and another time after cropping the forehead from the face. The test was repeated on two datasets: JAFFE and CK+.

Table 1 shows that the forehead is an essential part of the expression and must be included in the face to determine the correct expression. The forehead played an important role in determining expressions such as sad and angry.

**Table 1:** Increase of proposed accuracy method using image preprocessing techniques

	Datasets	
Accuracy	JAFFE	CK+
Before	94.84%	88.66%
After	96.71%	93.29%

#### Additional resizing

The last step was to resize the images with the detected face and the adjusted borders. This was done to ensure that the images with the detected faces were all equal in size, because the Viola–Jones algorithm detects faces with different border sizes due to the nature and size of the face and because of the border adjustment step. Therefore, the resizing had to be reimplemented to reach for standard image input.

After applying the border modification to the image, the size of the output image was recorded, and the minimum was noticed. The smallest size that was recorded was 112 × 92; therefore, all the images in all the datasets had to be of this size to ensure consistency and ensure the same number of features for all images. Table 1 displays how crucial image preprocessing is, because there was approximately a 2% and 5% increase in the accuracy of the Jaffe and CK+ datasets, respectively.

## Experimental results and discussion

Regarding FER, the two most famous and used validation methods to determine accuracy are the leave-one-out validation method and the K-fold validation method.

### Leave-one-out validation test

The leave-one-out validation test is used to determine the accuracy of the system by dividing the dataset into two categories: the testing set and the training set. The testing set contains one image, whereas the training set contains the rest of the dataset. The experiment is then repeated until all the images go through the system at least once, thus determining exactly what images are detected incorrectly out of the whole set [34].

For the datasets used, the number of experiments was 213 for JAFFE, 432 for CK+, and 1407 for the RaFD dataset. The results of the leave-one-out validation test on the three datasets are shown in Table 2.

**Table 2:** Proposed accuracy method using leave-one-out on the three datasets

Datasets	Leave-one-out
JAFFE	96.71%
CK+	93.29%
RaFD	99.72%

The accuracy of the JAFFE and RaFD datasets was higher than that of the CK+ one, because the numbers of images in each expression in those two datasets are equal, thus causing a fair training, whereas the CK+ dataset has a varying number of images for each expression. Also, the RaFD dataset showed the highest accuracy, because the training sample was extremely large compared with the other two datasets.

### K-fold validation test

The K-fold validation test is one form of the leave-one-out validation test that has several modifications applied to it. In the K-fold validation test, the dataset that has N images is divided into K sets. In each run, one of the K sets is used for testing, and the remaining sets are used for training. The process is repeated until all sets go through the system once. The only difference between the leave-one-out and K-fold validation tests is that the testing sample is larger for the K-fold one, thus decreasing the training set. Also, the randomness of the images being testing is larger, providing more-realistic results.

To validate the results as much as possible, several types of K-fold test were performed. The first test was the 10-fold validation test. In this test, the dataset was divided into 10 sets, and 10 iterations were applied to the system. The second test was the fivefold validation test, in which the dataset was divided into five sets of images. The sets in the fivefold validation test were larger than those in the 10-fold one. The last test was the twofold validation test, in which the dataset was divided into two halves: one for testing and the other for training. The results of the three discussed K-fold tests when applied to the three datasets are shown in Table 3.

**Table 3:** Proposed accuracy method using K-fold method

	Folds		
Datasets	10	5	2
JAFFE	98.10%	97.62%	90.10%
CK+	94.42%	93.49%	90.00%
RaFD	95.14%	95.10%	94.88%

The results of the 10-fold test were higher than those of the other two tests, because the training set was larger than that of the five fold and two fold tests. The CK+ accuracy was lower than those of the JAFFE and Radboud datasets, because CK+ is the only dataset that has a different number of images for each expression; therefore, the randomness in this dataset plays a large part in the accuracy achieved. The twofold test had the lowest accuracy of all the K-fold tests, because the system is not being trained enough, thus decreasing accuracy.

### Result overview

The overall results of performing the above-mentioned tests are summarized in Table 4. Table 4 shows that the model used has a high accuracy, more than 90%, under any test used and using any dataset. The results show that, if the proposed model is trained enough, the accuracy rises, as can be seen from the leave-one-out validation test. The CK+ dataset had the lowest accuracy among the datasets, because images in the CK+ dataset are not well distributed and vary in number.

**Table 4:** Proposed accuracy methods using different folds

Datasets	Methods			
	Leave-one-out	10 folds	5 folds	2 folds
JAFFE	96.71%	98.10%	97.62%	90.10%
CK+	93.29%	94.42%	93.49%	90.00%
RaFD	99.72%	95.14%	95.10%	94.88%

### Comparison

The last step is to compare the results obtained with results reported in previous work. Table 5 shows the results obtained by several researchers who developed FER systems. Each decided to implement a certain type of validation test different from the rest. In the last column of Table 5, the type of validation test performed is given.

**Table 5:** Proposed accuracy method compared with other techniques

Datasets	Method	Classification rate	Proposed method	Validation test
JAFFE	Patch-based Gabor [5]	92.30%	96.71%	Leave-one-out
	HOG+SVM [5]	94.30%		
	LDA + SVM [4]	95.71%		
	Boosted LBP + SVM [3]	79.80%	98.10%	10-folds
	LBP pyramid + SVM [1]	91.36%		
	LBP + Gabor filter [1]	92.38%		
	LBP + HOG + PCA + SVM [6]	87.60%	97.62%	5-folds
CK+	HOG+SVM [5]	88.70%	93.29%	Leave-one-out
	DNN [7]	93.20%	93.49%	5-fold
RaFD	HOG+SVM [20]	98.50%	99.72%	10-fold

Regarding the JAFFE database, the leave-one-out test result obtained surpassed the LDA + SVM [4] model by 1%, whereas the model that uses the same extraction and classification techniques with HOG and SVM [5] produced results that were less than those of the proposed model by 2.41%. The results of the K-fold tests also surpassed those of the previous models, especially the 10-fold model that had a huge accuracy difference of 5.70%.

The CK+ dataset also surpassed the previous models that used the same extraction and classification technique and could compete and surpass the performance of a deep neutral network [7], which is considered optimal in comparison with machine learning.

Regarding the RaFD dataset, although it is not very popular in the machine-learning domain, it was possible to compare it with a work that used the 10-fold test and was able to surpass it by more than 1%.

## Conclusions

An effective method of addressing the FER problem was proposed. Several steps were performed at the image preprocessing stage to ensure that the features attained from the face detected have a meaningful and proper contribution to the classification stage. The extra features at the side of the face (around the ears) and the ones at the bottom of the mouth (around the chin and neck) proved not to contribute any positive or useful information to the classification stage. Thus, it was necessary to crop them. In addition, it was essential for the mouth to be detected as a whole and not be cropped. Facial expressions were created as a result of muscle movements on the face. These subtle movements were detected by the HOG features, because HOG is sensitive to the shape of objects. The features attained are then used to train a network of binary linear SVMs. This network consists of three stages where the primary and first stage is made up of 21 SVMs that are all the binary combinations of the seven expressions. If one expression is dominant, then the first stage suffices. If two are dominant, then the second stage is used, and, if three are dominant, then the third stage is used. These multilevel stages reduce, as much as possible, the possibility of experiencing an error. Experimental results on three databases — JAFFE, CK+, and RaFD — show that the proposed system is competitive and has better performance compared with previous research results. Two different validation methods were used: the leave-one-out and K-fold tests. The percentages obtained from the leave-one-out test were 96.71%, 93.29%, and 99.72% for the JAFFE, CK+, and RaFD datasets, respectively. However, the percentages obtained from the 10-fold test (the most common fold test) were 98.10%, 94.42%, and 95.14%, respectively.

## Data Availability

Upon request.

## Abbreviations

CK+: The Extended Cohn-Kanade Dataset
FER: Facial Expression Recognition
HOG: Histogram of Oriented Gradients
JAFFE: Japanese Female Facial Expressions
LBP: Local Binary Patterns
RaFD: Radboud Facial dataset
SVMs: Support Vector Machines
